# Procalcitonin testing for diagnosis and short-term prognosis in bacterial infection complicated by congestive heart failure: a multicenter analysis of 4,698 cases

**DOI:** 10.1186/cc13181

**Published:** 2014-01-06

**Authors:** Weijia Wang, Xiuming Zhang, Na Ge, Jing Liu, Huimin Yuan, Peng Zhang, Wei Liu, Dongmei Wen

**Affiliations:** 1 Department of Laboratory Diagnosis, Sun Yat-Sen University Affiliated Zhongshan Hospital, Sun Yat-Sen University, No.2 Sunwen East Road, Zhongshan City 528403, Guangdong, China; 2 Department of Laboratory Diagnosis, Baotou Medical College, Inner Mongolia University of Science and Technology, No.31 Construction Road, Baotou City 014010, Inner Mongolia, China; 3 Department of Laboratory Diagnosis, Capital Medical University-Affiliated Chaoyang Hospital, Capital Medical University, 3.No.8 Worker's Stadium South Road, Beijing City 100020, Beijing, China; 4 Department of Laboratory Diagnosis, Nanfang Medical University-Affiliated Nanfang Hospital, Nanfang Medical University, No.1838 GuangZhou Avenue North Road, Guangzhou City 510515, Guangdong, China; 5 Department of Cardiology, Huazhong University of Science and Technology-Affiliated Union Hospital, No.1277 Libertador Road, Wuhan City 430022, Hubei, China

## Abstract

**Introduction:**

Procalcitonin (PCT) is a biomarker for the clinical diagnosis of bacterial infection that is more specific and earlier than fever, changes in white blood cell count, and blood cultures. Congestive heart failure is an important cause of endotoxin resorption from the intestine, which significantly increases PCT expression in noninfected patients with heart failure. The diagnostic performance and cut-off value of PCT in patients with bacterial infection complicated by congestive heart failure needs to be confirmed.

**Methods:**

A total of 4,698 cases from different cities in China, including those with different classes of congestive heart failure, bacterial infection, bacterial infection complicated by heart failure and healthy individuals, were chosen for the diagnostic value analysis of PCT and screening candidate predictors of mortality in subjects with bacterial infection complicated by congestive heart failure.

**Results:**

Patients with simple heart failure had significantly higher PCT levels than normal controls (*P* < 0.01), whereas patients with bacterial infection complicated by congestive heart failure had significantly higher PCT levels than those with simple infection (*P* < 0.01). Although it was useful for the diagnosis of infection (area under the receiver operating characteristic curve >80%), the positive predictive value of PCT decreased significantly with increasing severity of heart failure (*P* < 0.05), and the cut-off value of PCT concentrations for infection complicated by classes II, III and IV heart failure were 0.086, 0.192 and 0.657 μg/L, respectively. Heart failure degree, PCT level, and age were the candidate predictors of mortality in patients with bacterial infection complicated by congestive heart failure.

**Conclusions:**

These data suggest that complicated heart failure elevates the PCT level in patients with bacterial infection. Thus, the results of the PCT test must be analyzed correctly in consideration of the severity of heart failure. Close attention should be paid to cardiac function and PCT expression in aged patients with infection complicated by congestive heart failure.

## Introduction

The differential diagnosis between sepsis and noninfectious systemic inflammatory response syndrome is of great importance in the treatment of acutely ill patients because there might be an urgent need to change the antimicrobial regimens already administered or surgical eradication of the septic foci. The difficulty is aggravated further by the ambiguous results of the cultures of different biological fluids and by the rapid progression to multiple organ dysfunction [1]. Various serological indices have been applied to help this situation. Limited specificity has been demonstrated for C-reactive protein (CRP) and interleukin 6 (IL-6), for example, because their biosynthesis is triggered in infectious and noninfectious processes [2,3].

Procalcitonin (PCT) is a novel inflammatory marker of nonthyroid origin consisting of 116 amino acid residues. PCT levels are increased in the sera of patients with bacterial meningitis or sepsis [4-6], but they are not elevated in the setting of viral infections or autoimmune disorders [7,8]. Despite PCT levels’ being increased in the serum 6 hours after the intravenous administration of endotoxins in healthy volunteers [9], the exact locus of PCT production in sepsis is not known. Christ-Crain and colleagues [10] support the use of PCT assessments to decide whether to administer antibiotics to patients with infections of the lower respiratory tract. Researchers suggest that the PCT levels are normal if they are less than 0.1 μg/L and that PCT levels greater than 0.25 μg/L and above 0.5 μg/L are cutoffs for the consideration and initiation of antibiotic treatment, respectively [11,12]. However, the specific cutoff upon which this decision is based needs validation, particularly in other illnesses. Sandek *et al*. reported that the mean PCT level could reach 48 μg/L in negative cultures of blood, tracheal aspirates and urine of patients with more severe heart failure (for example, cardiogenic shock) [13]. Therefore, clinical doctors must analyze and estimate the PCT level correctly in patients with bacterial infections complicated by congestive heart failure.

## Materials and methods

The study protocol was approved by the Chinese Ethics Committee of Registering Clinical Trials. Written informed consent to be included in the study was provided by each patient.

### Demographics and pooled methodology

The samples from populations came from four cities in China: Guangzhou, Zhongshan, Wuhan and Beijing. The samples were drawn from among (1) 6,314 patients (age range, 18 to 75 years) admitted to hospitals in these cities because of heart failure or infection and (2) 446 healthy individuals undergoing health examinations. All four component data sets had comparable information available, including standard demographics, medical history and drug therapy, presenting symptoms and signs, physical examination, the results of serum chemistry tests, electrocardiography and the results of PCT and N-terminal pro-brain natriuretic peptide (NT-proBNP) tests. Glomerular filtration rate (GFR) was estimated using the modified diet in renal disease [14]. To determine the actual diagnosis, especially for heart failure patients, according to the guidelines issued by New York Heart Association (NYHA), two independent cardiologists and two independent physicians made the clinical diagnosis by reviewing all medical records (including echocardiographic data and laboratory results) pertaining to the patients. These records were cross-reviewed by clinical doctors in different research institutions. Hematological changes were not specific for bacterial infection, so the diagnosis of bacterial infection could be determined only by blood and secretion cultures. All of the final diagnoses were established based on clinical datasheets and additional information obtained during hospitalization.

The patient groups were classified as bacterial infection without heart failure (including septic shock in the advanced stage of infection), congestive heart failure without infection (heart failure only) and bacterial infection complicated by congestive heart failure (heart failure before bacterial colonization). Because it was hard to control mild and severe infections and the physical response to bacterial infection is varies significantly between patients, samples with positive pathogenic bacterial cultures were treated as a unified bacterial infection group. In order to avoid interference, patients with a history of viral infections and autoimmune disorders, which could slightly elevate the PCT level in serum, were excluded. In addition, samples in the control group were selected from healthy volunteers without hematological abnormalities (including white blood cell (WBC) count, CRP, IL-6 and NT-proBNP). Those participants whose test results were inconclusive and who had negative bacterial cultures and hematological abnormalities were also excluded. Even though infections can occur at any age, one study reported a transient increase in PCT expression in newborns and infants [15]. Inclusion of young patients (under 30 years old), who rarely experience heart failure, was avoided. Besides, owing to the difficulty in obtaining accurate classifications and the substantial variation of data in NYHA class I heart failure (asymptomatic heart failure), all samples were collected from patients with NYHA classes II, III or IV heart failure, who were classified according to guidelines set forth by the American College of Cardiology Foundation and the American Heart Association. Patients with severe heart failure and unavoidable renal dysfunction were evaluated by measuring GFR. Although we know that renal elimination of PCT is not a major mechanism for PCT removal from the plasma and that renal dysfunction does not greatly influence clinical diagnostic decisions [16], we still chose patients with high GFRs. From among the remaining complete and available data, we reached consensus on including 4,698 patients (Table 1).

**Table 1:** **Baseline demographics, results of physical examinations and laboratory tests and clinical diagnoses of the 4,698 study participants categorized with respect to population center**^**a**^

**Characteristics**	**Infection only**	**Heart failure only**	**Infection complicated by congestive heart failure**	**Healthy controls**
	**(*n*** **= 1,703)**	**(*n*** **= 1,364)**	**(*n*** **= 1,183)**	**(*n*** **= 448)**
Physical examination				
Age (mean ± SD)	51.1 ± 10.3	57.9 ± 14.7	58.5 ± 11.4	57.1 ± 18.3
Males (%)	51.7	48.3	49.4	50.0
Hypertension (%)	3.9	30.8	11.7	0
Chest pain (%)	2.4	33.7	18.6	0
Orthopnea (%)	0	13.9	29.7	0
Cough (%)	41.6	7.8	23.3	0
Fever (%)	84.6	0.4	77.1	0
Laboratory tests				
GFR (ml/min/1.73 m^2^), mean ± SD	71.7 ± 14.3	61.4 ± 18.2	64.1 ± 17.7	98.4 ± 5.5
WBC count (10^9^/L)	17.3 ± 9.7	7.4 ± 2.1	15.7 ± 8.0	7.8 ± 1.3
CRP (mg/L)	33.7 ± 19.6	11.7 ± 6.8	39.1 ± 18.4	4.7 ± 2.5
Positive blood culture (%)	39.3	0	22.7	0
Positive secretion/hydrothorax culture (%)	60.7	0	77.3	0
NT-proBNP, mean ± SD	196 ± 127	8,946 ± 4,969	5,116 ± 3,777	45 ± 11
IL-6, mean ± SD	21.3 ± 15.1	7.3 ± 3.5	19.4 ± 11.9	2.6 ± 0.9

^a^CRP, C-reactive protein; GFR, glomerular filtration rate; IL, interleukin; NT-proBNP, N-terminal pro-brain natriuretic peptide; WBC, white blood cell.

Follow-up for vital status among patients with bacterial infections complicated by heart failure was carried out by utilizing hospital records as well as contact with caregivers or patients, when appropriate, through 22 days from presentation (the mean hospital stay of patients with bacterial infections complicated by heart failure).

### Procalcitonin testing

PCT detection was conducted using a cobas E601 electrochemiluminescence immunoassay analyzer (Roche, Basel, Switzerland), the analytical performance of which has been confirmed to meet the requirements of the experiment (Additional file 1). The calibration solution (batch numbers 167488 and 16fq0068), analytical reagent (00162192) and quality control materials (16195300 and 16195400) were purchased from Roche.

### Comparative analysis of procalcitonin expression in different population groups

PCT levels of the specimens obtained from the healthy controls and patient populations with simple infection, simple heart failure and infection complicated by heart failure were compared and analyzed to identify the variation patterns of PCT expression in different populations. We examined the differential expression of PCT in patients with simple heart failure relative to the controls and that in patients with bacterial infection complicated by heart failure relative to simple infection.

### Comparative analysis of diagnostic performance of procalcitonin using receiver operating characteristic curves

A comprehensive analysis was conducted on the results of PCT detection with specimens from patients with simple bacterial infections and those with infections complicated by heart failure. The true-positive diagnostic cutoff of PCT was set to 1.0, and the true-negative diagnostic cutoff was set to 0.0 with 95% CI. The diagnostic performance of PCT for simple bacterial infections and infections complicated by heart failure was evaluated, and the cutoffs were determined.

### Short-term prognosis for patients with bacterial infections complicated by congestive heart failure

Of 1,182 patients with infections complicated by congestive heart failure, 134 died in the hospital or in other medical centers within 22 days. The candidate predictors of mortality in patients with bacterial infections complicated by heart failure were screened by Cox regression analysis using sex, age, class of cardiac function, body temperature and commonly used hematological parameters (for example, levels of PCT, WBC count, CRP and IL-6) as independent variables. Although BNP and NT-proBNP are biomarkers widely used in making a heart failure diagnosis, we found in our earlier work that they are confounded by ischemic diseases, such as cerebral infarction [17]. So, BNP and NT-proBNP were excluded as variables in this study.

### Statistical analysis

Data analyses were carried out using SPSS version 19.0 software (SPSS, Chicago, IL, USA). A test for normal distribution was done using the Kolmogorov–Smirnov method. Mean values that did not follow a normal distribution were compared using the Kruskal–Wallis H statistic. For pairwise comparisons, the level of significance was adjusted using the Bonferroni method. Diagnostic tests were assessed by receiver operating characteristic (ROC) curve analyses. Predictors of mortality were screened out by Cox regression analysis using a forward stepwise conditional method. *P* < 0.05 was considered significant.

## Results

### Expression patterns of procalcitonin in different populations

**Figure 1:**
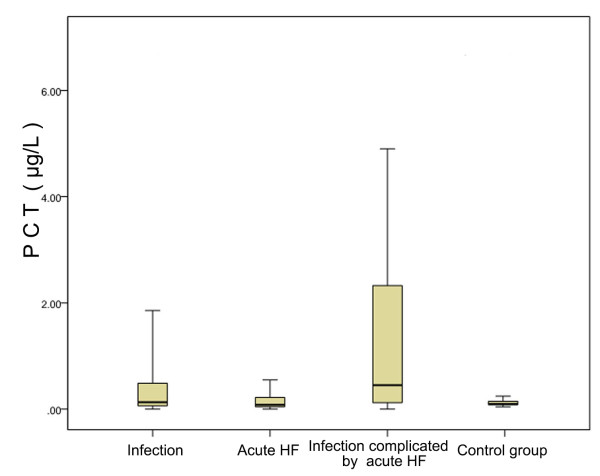
**Differential expression of procalcitonin in different populations.** HF, Heart failure; PCT, Procalcitonin. Boxes represent interquartile range, while whiskers the 5th and 95th percentiles in each category.

PCT expression showed significant differences among the four population groups (*P* < 0.05) (Table 2). PCT levels were significantly higher in patients with simple heat failure than in those in the control group (*P* < 0.05), verifying the notion that heart failure can elevate PCT levels [13]. Patients with bacterial infections complicated by congestive heart failure had significantly higher PCT levels than those with simple bacterial infections, suggesting that heart failure may influence a PCT-based diagnosis of infection, as shown in Figure 1.

**Table 2:** **Comparison of procalcitonin expression according to population**^**a**^

**Group**	**PCT**						
	**Median**	**Interquartile range**	**Mean rank**	**χ**^**2 **^**(overall)**	***P *** **(overall)**	**χ**^**2 **^**(group)**	***P *** **(group)**
Simple infection (1)	0.28	0.06 to 0.49	1,661.01	446.9	0.00	(12) 52.7	(12) 0.00
						(13) 233.8	(13) 0.00
						(14) 77.6	(14) 0.00
Simple heart failure (2)	0.13	0.05 to 0.22	1,288.63			(23) 252.9	(23) 0.00
						(24) 9.10	(24) 0.00
						(34) 205.7	(34) 0.00
Infection complicated by congestive heart failure (3)	0.45	0.12 to 2.59	2,232.60				
Healthy control (4)	0.04	0.05 to 0.12	996.42				

^a^PCT, Procalcitonin.

### Diagnostic performance of procalcitonin for simple bacterial infections and infections complicated by congestive heart failure

**Figure 2:**
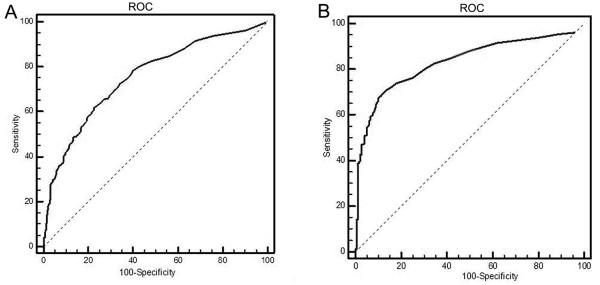
**Receiver operating characteristic curve for the procalcitonin diagnosis of simple infection and infection complicated by congestive heart failure. (A)** Diagnostic curve of procalcitonin (PCT) for simple bacterial infections. The area under the receiver operating characteristic curve shown is above 80%, which means PCT still has the diagnostic value for simple bacterial infection and infection complicated by congestive heart failure. **(B)** Diagnostic curve of PCT for infection complicated by congestive heart failure. ROC, Receiver operating characteristic curve. The dashed line is the baseline that result from random classification.

**Figure 3:**
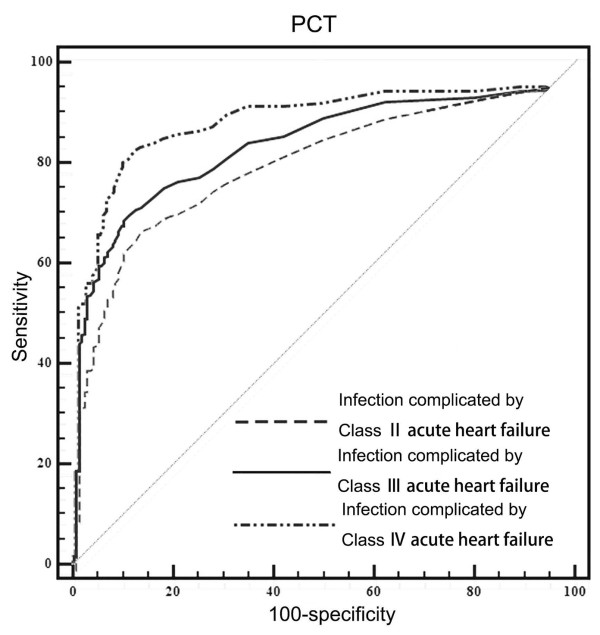
**Receiver operating characteristic curve for procalcitonin-based diagnosis of infections complicated by different classes of heart failure.** As depicted, procalcitonin (PCT) had high areas under the receiver operating characteristic curve in each heart failure group. However, the best cutoff values for each group were different.

In accordance with the manufacturer’s instructions (electro- chemiluminescence immunoassay analyzer, Roche Cobas E601, Basel, Switzerland) and data previously published in the literature [5], we chose 0.1 μg/L as the cutoff for comparing the diagnostic value of PCT among different populations. PCT was used for the diagnosis of infections, so a comparative analysis was undertaken between patients with simple bacterial infections and those with infections complicated by congestive heart failure. At the fixed PCT level of 0.1 μg/L, the diagnostic sensitivity of PCT was significantly higher for infections complicated by heart failure than for simple infections, whereas the corresponding positive predictive value of PCT was significantly lower for the former than the latter population (Table 3). The positive predictive value of PCT decreased significantly with increasing severity of heart failure (*P* < 0.05). Nevertheless, PCT has a certain diagnostic value for simple bacterial infections and infections complicated by heart failure (NYHA classes II to IV) (area under the ROC curve (AUC) >80%) (Figure 2). The diagnostic cutoffs of PCT for patients with class II, III, or IV heart failure increased significantly with the severity of heart failure (0.086, 0.192 and 0.657 μg/L, respectively) (Figure 3).

**Table 3:** Comparison of procalcitonin diagnosis between simple infection and different classes of infection complicated by congestive heart failure

**Class**	**Cutoff (μg/L)**	**Sensitivity**	**Youden index**	**Positive predicative value**	**Negative predicative value**	**Accuracy**	**Z-score**	***P***
Simple infection	0.1	56.3	0.284	95.1	14.7	57.8	11.345	<0.05
Infection complicated by NYHA class II heart failure	0.1	76.6	0.487	90.9	45.7	75.6	20.232	<0.05
Infection complicated by NYHA class III heart failure	0.1	78.4	0.505	87.6	57.1	76.6	20.168	<0.05
Infection complicated by NYHA class IV heart failure	0.1	87.2	0.593	68.6	89.0	78.3	16.518	<0.05

^a^NYHA, New York Heart Association.

### Short-term prognosis in patients with infections complicated by congestive heart failure

**Figure 4:**
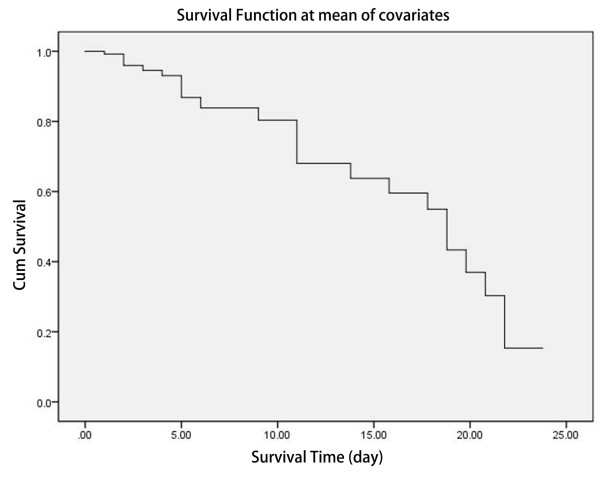
**Cumulative survival function at mean of covariates for survival analysis in infections complicated by congestive heart failure during 22 days hospitalization.** The horizontal axis is survival time (days), and the vertical coordinate is the probability of survival at the corresponding times. This is a decline curve, which means the steeper the curve is, the shorter the survival time will be. The slope indicates the death rate.

Sex, age, body temperature; PCT, WBC, CRP and IL-6 levels; and class of cardiac function were used as the independent variables for Cox regression analyses and were screened using the conditional forward stepwise method. Three factors were screened and included in the equation: age, PCT level and class of cardiac function. The χ^2^ test of the model produced χ^2^ = 73.393 (*P* < 0.01), which means there were statistical differences in this model. The impact factors included in the equation are shown in Table 4. Age, PCT level and cardiac function were the candidate predictors of mortality, with relative risk (RR) of 1.061 times (1.006 to 1.119), 1.110 times (1.053 to 1.170) and 2.719 times (1.319 to 5.605), respectively. The cumulative survival function at the mean of covariates is shown in Figure 4.

**Table 4:** **Candidate predictors of mortality in patients with infections complicated by heart failure**^**a**^

**Factors**	**β**	**SE**	**Wald**	***df***	**Significance**	**RR**	**RR 95.0% CI**
Age	0.059	0.027	4.734	1	0.030	1.061	1.006 to 1.119
Cardiac function class	1.000	0.369	7.343	1	0.007	2.719	1.319 to 5.605
PCT level	0.104	0.027	15.047	1	0.000	1.110	1.053 to 1.170

^a^PCT, Procalcitonin; RR, Risk ratio. With each 1-year increase in age, the possibility of death increases by 1.061 times. In patients with PCT <0.1, the possibility of death increases by 1.11 times compared with those with PCT >0.1. With each one-class increase in cardiac function, the possibility of death increases by 2.719 times. Data are derived from 134 patients.

## Discussion

PCT is a peptide precursor of calcitonin which has commonly been used for the early diagnosis of sepsis, for the differential diagnosis between bacterial and viral infections and as a guide for the use of antibiotics in clinical treatment [18]. However, the results of our retrospective analysis show that patients with simple heart failure had significantly higher levels of PCT than the controls, whereas patients with bacterial infections complicated by heart failure had significantly higher levels of PCT than those with simple bacterial infections. The diagnostic performance of PCT in patients with bacterial infections complicated by heart failure needs to be reconsidered, and the cutoff value should be confirmed.

Sandek *et al*. [13] found that heart failure interferes with the PCT-based diagnosis of infection. They stated that the diagnostic cutoff of PCT should be established in various disease states. Despite the necessity of the diagnostic cutoff, some essential difficulties remain, such as the lack of specific standards and exclusion standards, no clinical manifestation differences between systemic and nonsystemic infections and difficulty in experimental control conditions. To ensure that the experimental conditions were consistent in our present multicenter study, the samples with consensus diagnosis were collected and processed, and we performed the tests using reagents from the same manufacturer. The laboratories that took part in our present study (Roche Cobas E601, Basel,Switzerland) accepted and went through the external quality assessment initiated by National Ministry of Health Department, and the precision and accuracy of detection have been verified to comply with the requirements of the clinical test.

The findings in this study suggest that heart failure may interfere with PCT expression. However, we found that PCT detection was still useful in the diagnosis of bacterial infections complicated by heart failure (AUC >80%) and that the diagnostic sensitivity increased with the severity of heart failure. This finding does not prove that PCT is conducive to making the diagnosis, but it does indicate that PCT levels are increased in patients diagnosed with infection complicated by heart failure, thus increasing the diagnostic sensitivity. This factor is also a main reason why the positive predictive value of PCT decreases with the severity of heart failure. Heart failure can affect the PCT level, so the diagnostic cutoff value of PCT needs to be altered accordingly. In this study, we have shown that the PCT level increased significantly with increasing severity of heart failure in noninfected patients and that the cutoff concentration of PCT was up to 0.657 μg/L in patients with simple NYHA class IV heart failure. These observations suggest that clinical doctors should consider the severity of heart failure during the diagnosis of a specific infection. The PCT level in some patients of the infection group in this study was not very high. There might be two reasons affecting the PCT level in patients with bacterial infection. First, the PCT level in patients with Gram-positive bacterial infections is much lower than that in patients with Gram-negative bacterial infections [19]. Second, inflammatory mediators such as IL-1β, tumor necrosis factor α and IL-6, which could elevate serum PCT levels in some elderly patients, especially those with poor immune response and those with local infections in early stages, might not be induced as soon as bacterial colonization [20,21].

In patients with heart failure, infections (especially pulmonary infections) can cause continuous progression of heart failure by complementary pathogenic mechanisms. In contrast, cases of patients who die as a result of simple heart failure or myocardial infarction are rare. In this short-term prognostic analysis, the three infection-screening markers commonly used in medical laboratories (WBC count, CRP and IL-6) were not chosen as candidate predictors of mortality.

These three factors may lack specificity for the diagnosis of infections complicated by heart failure. In particular, a considerable number of the patients with infections but no fever had no increases in (WBC count, CRP and IL-6) [22]. CRP acts as a stress protein and cannot be used to prove that infection is present. Despite reports on its diagnostic application, IL-6 lacks specificity in the diagnosis of viral and bacterial infections [23]. Cardiac function declines to varying degrees as people age. However, the decline in cardiac function inevitably decreases in the circulatory function of the whole body and promotes the constant sorption of endotoxins into the bloodstream, leading to an elevation of PCT level. Additionally, functional decline induces a series of infections, which has a synergistic promotion effect on the elevation of PCT expression. Therefore, close attention should be paid to patient age, severity of heart failure and PCT level in the treatment of patients with bacterial infections complicated by heart failure.

## Conclusion

Heart failure is a common factor that interferes with PCT diagnostic value in patients with bacterial infections. The laboratory data of patients with bacterial infections complicated by congestive heart failure should be analyzed comprehensively, and a complete clinical nursing program should be adjusted. At present, the clinical diagnosis of infections is primarily experience-based, and specific classification criteria are lacking. The diagnosis and classification of early infection with collaborative applications of effective infection markers remain the focus of clinical research.

## Key messages

• PCT level increases significantly with increasing severity of heart failure in noninfected patients.

• PCT has a certain diagnostic value for simple infections and infections complicated by heart failure (NYHA classes II to IV) (AUC >80%); however, the positive predictive value of PCT decreases with the severity of heart failure.

• The diagnostic cutoffs of PCT for patients with NYHA classes II –to IV heart failure increased significantly with the severity of heart failure (0.086, 0.192 and 0.657 μg/L, respectively).

• Age, PCT level and cardiac function were the candidate predictors of mortality, with RRs of 1.061 times (95% CI, 1.006 to 1.119), 1.110 times (1.053 to 1.170) and 2.719 times (1.319 to 5.605), respectively.

• In order to prolong survival time, the laboratory data of patients with infections complicated by congestive heart failure should be analyzed comprehensively, and a complete clinical nursing program should be adjusted accordingly.

## Abbreviations

CRP: C-reactive protein; GFR: Glomerular filtration rate; IL-6: Interleukin 6; NT-proBNP: N-terminal pro-brain natriuretic peptide; PCT: Procalcitonin; WBC: White blood cell.

## Competing interests

The authors have no competing interests to declare.

## Authors’ contributions

WW and XZ developed the study design and coordinated its implementation. NG, JL, HY, WL and PZ were responsible for patient recruitment as well as data collection, and they carried out the statistical analysis. DW participated in the interpretation and discussion of the results and drafted and revised the manuscript. All authors read and approved the final manuscript.

## Supplementary Material

Additional file 1 Verification of the precision and accuracy of PCT detection. Click here for file
