# Supplementary material for: Procalcitonin testing for diagnosis and short-term prognosis in bacterial infection complicated by congestive heart failure: a multicenter analysis of 4,698 cases
Source: Crit Care. 2014 Jan 6;18(1):R4. doi: 10.1186/cc13181 (PMC4056105; doi:10.1186/cc13181)
Supplement: Additional file 1 — Verification of the precision and accuracy of PCT detection. [file cc13181-S1.doc]

**Verification of the precision and accuracy of PCT detection**

**Materials and methods**

PCT detection was conducted using a Roche Cobas E601 Electrochemiluminescence Immunoassay Analyzer (Roche, Basel, Switzerland). The calibration solution (batch numbers 167488 and 160068), analytical reagent (00162192), and quality control (QC) materials (16195300 and16195400) were purchased from Roche.

The precision and accuracy of detection were verified by following Document EP15-A2 from the Clinical and Laboratory Standards Institute [1]. In accordance with the instrument manual, the experimental concentrations of PCT were chosen to be as close to the values of the analytes evaluated by the manufacturer: 0.55, 10.46, 0.10, 0.48 and 56.84 μg/L. Except for the last two concentrations for QC materials, the selected concentrations of high-level test specimens were formulated with low-level serum and stored at −70°C until analyses. The theoretical concentrations of the prepared specimens were obtained as the arithmetic means of five continuous measurements. Then, a specimen of each concentration was divided into 22 aliquots followed by the daily assay of four aliquots for 5 consecutive days. The two levels of assigned-value calibrators with different batch numbers were assayed thrice. The mean values of measurement results were compared with the target value to calculate the relative deviation (RD). RD ≤ 1/2 total allowable error was used to evaluate the acceptability of the accuracy of the instrument.

**Results**

The precision of PCT detection with different concentrations of specimens is shown in Table 1. Within the selected range of concentration, the manufacturer stated that the batch coefficient of variation (CV) of PCT was 1.10–7.11%, and that the total CV of PCT was 1.65–8.73%. The experimental results were all less than those reported by the manufacturer, and complied with the requirement of the laboratory quality target CV (i.e., < 10%). The accuracy of PCT detection was obtained by repeated measurements of a PCT calibrator from different batches and calculation of the bias. The RD of PCT measurement results to the assigned values of reference materials was (2.17±1.21)%, i.e., < 5.0%.

**Table 1 Precision of Roche Cobas E601 system for PCT detection**

| Item | Results | | | | |
| --- | --- | --- | --- | --- | --- |
| Specimen | PC1 | PC2 | PC3 | PC4 | PC5 |
| Mean | 0.55 | 10.46 | 0.10 | 0.48 | 56.84 |
| Batch SD (μg/L) | 0.01 | 1.24 | 0.01 | 0.01 | 3.49 |
| Batch CV (%) | 2.61 | 1.44 | 3.57 | 2.87 | 1.33 |
| Overall SD (μg/L) | 0.03 | 1.35 | 0.01 | 0.02 | 4.19 |
| Overall CV (%) | 2.94 | 1.55 | 3.59 | 3.09 | 1.93 |

**Conclusion**

The instrument conditions complied with the requirements of the clinical test.

**Reference**

1. NCCLS.EP15-A User demonstration of performance for precision and accureacy, approved guideline. *Wayne* PA:NCCLS,2001
